# Persistence of schistosomal transmission linked to the Cavu river in southern Corsica since 2013

**DOI:** 10.2807/1560-7917.ES.2018.23.4.18-00017

**Published:** 2018-01-25

**Authors:** Lauriane Ramalli, Stephen Mulero, Harold Noël, Jean-Dominique Chiappini, Josselin Vincent, Hélène Barré-Cardi, Philippe Malfait, Guillaume Normand, Florian Busato, Vincent Gendrin, Jean-François Allienne, Judith Fillaux, Jérôme Boissier, Antoine Berry

**Affiliations:** 1 Santé publique France, Regional office of the French national public health agency in Provence-Alpes-Côte d’Azur and Corsica, Saint-Maurice, France; 2 European Programme for Intervention Epidemiology Training (EPIET), European Centre for Disease Prevention and Control (ECDC), Stockholm, Sweden; 3 Both authors contributed equally to the study and manuscript writing; 4 French National Centre for Scientific Research (CNRS) (UMR 5244), University of Perpignan, Perpignan, France; 5 Santé publique France, French national public health agency, Saint-Maurice, France; 6 Regional Health Agency of Corsica, Ajaccio, France; 7 Environnement Agency of Corsica (Office de l’environnement de Corse), Corsica, France; 8 Urology department, Ormeau polyclinic, Tarbes, France; 9 Internal Medicine, Bigorre Hospital, Tarbes, France; 10 Infectious disease department, Nord Franche-Comté Hospital, Trévenans, France; 11 Department of Parasitology-Mycology, CHU Toulouse, Toulouse, France; 12 Both authors contributed equally to the study; 13 Physiopathology Centre of Toulouse-Purpan, Toulouse University, CNRS, INSERM, UPS, Toulouse, France

**Keywords:** Schistosomiasis, parasitic diseases, bulinus, outbreaks, Corsica, France

## Abstract

Seven cases of urogenital schistosomiasis occurred in Corsica in 2015 and 2016. The episodes were related to exposure to the same river and involved the same parasite strain as an outbreak with 106 cases in summer 2013. The connection calls for further investigations on the presence of an animal reservoir and the survival of infested snails during winter. However, recontamination of the river from previously infected bathers remains the most likely hypothesis.

Since 2015, seven cases of urogenital schistosomiasis reported exposure to the Cavu river in southern Corsica. They had no history of contact to fresh water in endemic areas. Here, we describe the cases indicating persistent schistosomal transmission linked to this river since 2013. To date, no contamination has been related to an exposure in 2017, but cases might arise in the coming months considering the long parasitic cycle in humans. Therefore, physicians in France and elsewhere should test possible clinical cases, regardless of the year of exposure to the Cavu river. 

## Case description

In 2015 and 2016, five cases of urogenital schistosomiasis linked to exposure to the Cavu river in 2015 were notified to regional health authorities. An autochthonous urogenital schistosomiasis case was defined as a person with serological evidence of schistosomiasis or *Schistosoma* eggs in urine, and no history of contact with fresh water in known endemic areas. A case was classified as confirmed when presenting *Schistosoma* eggs at urine or biopsy examination. We classified a case as probable when the person either presented one positive serological test and a positive Western blot, or two positive serological tests and no Western blot. A possible case was a person presenting one isolated positive serological test or two discordant serological tests.

The first probable case was detected at the end of 2015 (Table, Case 1) and described in a previous article [1]. In March 2016, based on the results from different serological tests for schistosomiasis, two members of another family were classified as probable cases (Table, Family 1). Cases 1, 2 and 3 reported swimming in the Cavu River in August 2015 in two different locations (Table). One location corresponded to a main focus of *Schistosoma* infections previously identified in an outbreak in 2013 [2], while the other was a location not known as a transmission area.

**Table:** Demographics, clinical manifestation, laboratory results and place of exposure for cases of autochthonous urogenital schistosomiasis contracted in the Cavu River, Corsica, France, 2015 or 2016 (n = 7)

	Case 1	Family 1		Family 2		Family 3	
		Case 2	Case 3	Case 4	Case 5	Case 6	Case 7
**Demographics**							
Adult (≥ 18 years)	Yes	No	Yes	No	Yes	Yes	Yes
Resident of Corsica	No	No	No	No	No	No	No
**Clinical manifestation**							
Symptoms present	Yes	No	No	No	Yes	Yes	No
Symptoms	Abdominal pain, fatigue	NA	NA	NA	Dysuria, pollakiuria, renal colic	Dysuria, pollakiuria, haematuria	NA
Onset	Aug 2015	NA	NA	NA	End of 2016	End of 2016	NA
**Laboratory results**							
Date of diagnosis	Oct 2015	Mar 2016	Mar 2016	Feb 2017	Feb 2017	Sep 2017	Sep 2017
ELISA^a^	4.67	2.54	1.81	0.91	0.22	2.10	0.9
IHA^b^	320	320	160	80	0	160	160
Western blot (KDa bands)^c^	30-34	22–24	22–24, 30–34	22–24, 30–34	22–24, 30–34	22–24, 30–34	22–24, 30–34
Eggs in urine or bladder biopsy	Negative	Negative	Negative	Positive^d^	Positive^e^	Positive^d,e^	Negative
**Case classification**	Probable	Probable	Probable	Confirmed	Confirmed	Confirmed	Possible
**Exposure to the Cavu river**							
Year	2015	2015	2015	2015 and 2016	2015 and 2016	2016 and 2017	2016 and 2017
Period	30 Jul–11 Aug	15–30 Aug	15–30 Aug	30 Jul–14 Aug	30 Jul–14 Aug	15–30 Jul	15–30 Jul
**Reported swimming sites in the Cavu river**							
3 piscines^f^	No	No	No	Yes	Yes	Yes	Yes
Outdoor activity park^g^	Yes	No	No	Yes	Yes	Yes	Yes
Bridge Mulinu di Conca^h^	No	Yes	Yes	No	No	No	No

ELISA: enzyme-linked immunosorbent assay; IHA: indirect haemagglutination assay; NA: not applicable.

^a^ Bordier Affinity Products, Crissier, Switzerland. Positive if ≥ 1 (DO sample/DO control).

^b^ Fumouze Diagnostics, Levallois-Perret, France. Positive if titre ≥ 1/160.

^c^ SCHISTO II Western blot IgG, LDBIO Diagnostics, Lyon, France. Positive with observation of bands at 22–24 kDa or 30–34 kDa [6].

^d^ *Schistosoma* eggs identified in urine in microscopic examination.

^e^ *Schistosoma* eggs identified in bladder biopsy.

^f^ Latitude 41°43’56–66”N, longitude 9°17’38–11”E.

^g^ Latitude 41°43’22–10”N, longitude 9°18’0–39”E.

^h^ Latitude 41°42’17–03”N, longitude 9°20’05–02”E [5].

In February 2017, two members of another family were classified as confirmed cases upon identification of *Schistosoma* eggs in urine or bladder biopsy. They reported bathing in the Cavu, in both 2015 and 2016 (Table, Family 2). However, calcified eggs observed in the bladder wall of Case 4 suggest an old infection, probably attributable to exposure in 2015.

In September 2017, a new confirmed case was diagnosed upon identification of *Schistosoma* eggs in urine and biopsy of a bladder wall thickening, with a 9-month gross haematuria (Table, Family 3, Case 6). The patient reported exposure to the Cavu in summer 2016 and 2017; however, with only four weeks between the 2017 exposure and the diagnosis, the infection presumably occurred in 2016. Screening of the case’s family members (n = 7), who shared the same exposure, identified one additional possible case (Table, Case 7).

*Schistosoma* eggs from Case 4 and Case 6 were genotyped as previously described [2]. The eggs presented the same genotype, carrying the nuclear internal transcribed spacer (ITS) region from *S. haematobium* and the mitochondrial cytochrome oxidase subunit 1 (cox1) gene from *S. bovis*. All mitochondrial genes presented the Sb2 haplotype: this was the most common haplotype in the 2013 outbreak and is identical to the type found in people infected in West Africa [2].

## Environmental surveillance

Since 2014, *Bulinus truncatus* snails, the intermediate host of *S. haematobium*, have been collected weekly from the Cavu River from June to September, when water temperatures are optimal for schistosomal transmission. In 2014 and 2015, respectively 3,544 and 1,965 snails tested negative using microscopic techniques to observe the shedding of *Schistosoma* cercariae. Using a more sensitive technique relying on PCR amplification of the *Schistosoma* Dra1 repeat sequence, the 3,453 and 5,364 specimens collected in 2016 and 2017, respectively, also tested negative [3].

## Discussion

In 2013, urogenital schistosomiasis re-emerged in Europe, with 106 cases linked to exposure to the Cavu river in southern Corsica [4-7]. The parasite strain was a human-bovine hybrid *Schistosoma* *(S. haematobium – S. bovis)* of Senegalese origin [2]. Following this event, we developed surveillance for human cases and infected aquatic snail hosts to detect and control schistosomal transmission. Autochthonous urogenital schistosomiasis became notifiable in France in 2016.

The identification of three episodes of transmission over a 4-year period (2013, 2015 and 2016) and the close genetic relationship among the isolated parasites confirmed that schistosomes were maintained in a transmission cycle in or near the Cavu River in Corsica. Although cases with disputed serological evidence of infection (because of weak positivity) were reported among European travellers exposed to the Cavu River in 2014, no cases identified among French tourists or Corsican residents could be linked to the same year of exposure [8,9,10].

The presence of one or a combination of the following factors would lead to an ongoing transmission of schistosomiasis in Corsica linked to the Cavu River: the survival of infested snails over the winter, the presence of an animal reservoir along the river, or repeated bathing in this river by a human reservoir.

Whether or not infested snails survive in the Cavu River over the winter period is still uncertain. However, because the period between the first identified transmission in 2013 and the last in 2016 is longer than the expected lifetime of a snail, the survival of infested snails alone would not explain schistosomal transmission over a 4-year period.

The presence of a reservoir in livestock is unlikely, considering negative results from a previous *Schistosoma* seroprevalence study in cows and goats in southern Corsica (n = 3,479). However, in light of the persistent transmission, the presence of a murine reservoir cannot be excluded. A screening study in rodents in 2015 yielded negative test results for *Schistosoma* infestation but was too small (n = 21) to be conclusive [2]. 

The persistence of a human reservoir contaminated in the Cavu river appears possible, despite the screening and treatment efforts carried out in 2014 by health authorities and physicians [4]. Detection of infected individuals is challenging, as 66% of persons infected in the 2013 outbreak remained asymptomatic, and the median time to first symptom onset was 30 weeks [4]. In addition, this series of notified cases shows that the positivity threshold set for the first-line screening tests (ELISA and indirect haemagglutination assay) can miss confirmed cases. Both screening tests for Cases 4 and 5 were negative more than 6 months after their last exposure, while *Schistosoma* eggs were detected through the microscopic examination of bladder biopsy or urine. Conversely, screening results well above the test threshold, associated with a positive confirmatory test (as in Cases 1 to 3), are strongly supportive of the diagnosis of schistosomiasis.

Environmental surveillance aims to detect at an early stage the presence of the parasite in the Cavu river in the summer season in order to implement rapid measures for limiting schistosomal transmission. Although several humans were infected in 2015, microscopic observation of molluscs did not detect the parasite. The PCR-based method used since 2016 might be more sensitive for detecting *Schistosoma* genetic material in both emitting and non-emitting molluscs. However, it did not detect any infested snails in 2016, when several persons were infected. This suggests a low level of infestation among river snails, and therefore low transmission of schistosomiasis. Environmental surveillance relies on weekly sampling of *B. truncatus* snails, which could miss the occasional infested specimen; however, only one infected snail is enough to contaminate several humans, or continue the cycle of transmission.

Given the probably limited transmission between 2015 and 2016 and the importance of the Cavu river as a tourism site, screening all individuals who report exposure to the river, regardless of year of exposure and presence of symptoms, is unlikely to be cost-effective in identifying a persistent human reservoir.

## Conclusion

Even if the most likely hypothesis for the maintenance of the parasite in the Cavu river is re-seeding by human hosts, other environmental factors cannot be yet ruled out. Research into the survival of infested snails during winter and a possible rodent reservoir along the river are necessary to evaluate the risk of ongoing transmission of urogenital schistosomiasis in the Cavu river. Techniques to detect the presence of the parasite directly in the water of the river are needed [11]. In the meantime, improving awareness among tourists and local residents exposed to the river and screening recommendations for physicians are fundamental as further cases could occur or become symptomatic. In order to increase the detection of cases and avoid any infected individuals remaining undiagnosed, diagnostic methods should be improved and the diagnostic strategy revised accordingly.

To better target information and communication strategies, Santé publique France and the regional health authority will conduct a study among general practitioners in Corsica on knowledge, attitudes and practices regarding schistosomiasis. In parallel, awareness campaigns directed at national and international tourists and local residents about the risk of contracting schistosomiasis should be pursued and reinforced. The maintenance of schistosomes on Corsica should alert other regions in Europe with environmental conditions favourable to urogenital schistosomiasis transmission. Persons exposed to the Cavu river presenting symptoms compatible with urogenital schistosomiasis should be tested, regardless of the year of exposure.
